# Retraction Note: Alcohol consumption and hormonal alterations related to muscle hypertrophy: a review

**DOI:** 10.1186/1743-7075-11-43

**Published:** 2014-09-24

**Authors:** Antonino Bianco, Ewan Thomas, Francesco Pomara, Garden Tabacchi, Bettina Karsten, Antonio Paoli, Antonio Palma

**Affiliations:** Sport and Exercise Sciences Research Unit, University of Palermo, Via Eleonora Duse, 2, 90146 Palermo, Italy; MEDEOR Research Institute, Via Emilio Salgari, 69, 90147 Palermo, Italy; Department of Sciences for Health Promotion and Mother-Child Care “G. D’Alessandro”, University of Palermo, Via del Vespro 133, 90127 Palermo, Italy; Life and Sports Science, University of Greenwich, Central Avenue, ME4 4 TB Chatham Maritime, UK; Department of Biomedical Sciences (DSB), University of Padova, Via Marzolo 3, 35131 Padova, Italy

## Retraction

This article [1] has been retracted by the Editors due to extensive overlap with previously published work [2]. The Editors apologise for any inconvenience caused.

## References

[1] Bianco A, Thomas E, Pomara F, Tabacchi G, Karsten B, Paoli A, Palma A (2014). Alcohol consumption and hormonal alterations related to muscle hypertrophy: a review. Nutrition & Metabolism.

[2] *Alcohol - Scientific Review on Usage, Dosage, Side Effects*. *Examine.com* at http://examine.com/supplements/Alcohol/ Examine.com at

